# Patient outcomes following emergency admission to hospital for COVID-19 compared with influenza: retrospective cohort study

**DOI:** 10.1136/thoraxjnl-2021-217858

**Published:** 2022-07-27

**Authors:** Thomas Woodcock, Geva Greenfield, Ajit Lalvani, Azeem Majeed, Paul Aylin

**Affiliations:** 1 Department of Primary Care and Public Health, Imperial College London, London, UK; 2 School of Public Health, Imperial College London, London, UK; 3 NIHR Health Protection Research Unit in Respiratory Infections, Imperial College London National Heart and Lung Institute, London, UK

**Keywords:** COVID-19, viral infection, respiratory infection

## Abstract

**Background:**

We examine differences in posthospitalisation outcomes, and health system resource use, for patients hospitalised with COVID-19 during the UK’s first pandemic wave in 2020, and influenza during 2018 and 2019.

**Methods:**

This retrospective cohort study used routinely collected primary and secondary care data. Outcomes, measured for 90 days follow-up after discharge were length of stay in hospital, mortality, emergency readmission and primary care activity.

**Results:**

The study included 5132 patients admitted to hospital as an emergency, with COVID-19 and influenza cohorts comprising 3799 and 1333 patients respectively. Patients in the COVID-19 cohort were more likely to stay in hospital longer than 10 days (OR 3.91, 95% CI 3.14 to 4.65); and more likely to die in hospital (OR 11.85, 95% CI 8.58 to 16.86) and within 90 days of discharge (OR 7.92, 95% CI 6.20 to 10.25). For those who survived, rates of emergency readmission within 90 days were comparable between COVID-19 and influenza cohorts (OR 1.07, 95% CI 0.89 to 1.29), while primary care activity was greater among the COVID-19 cohort (incidence rate ratio 1.30, 95% CI 1.23 to 1.37).

**Conclusions:**

Patients admitted for COVID-19 were more likely to die, more likely to stay in hospital for over 10 days and interact more with primary care after discharge, than patients admitted for influenza. However, readmission rates were similar for both groups. These findings, while situated in the context of the first wave of COVID-19, with the associated pressures on the health system, can inform health service planning for subsequent waves of COVID-19, and show that patients with COVID-19 interact more with healthcare services as well as having poorer outcomes than those with influenza.

**WHAT IS ALREADY KNOWN ON THIS TOPIC**

- Despite initial assumed similarities in clinical features of COVID-19 and influenza, they have different clinical manifestations and clinical outcomes; however, published evidence is bounded to Spring 2020 and mainly come from distinct medical centres.

**WHAT THIS STUDY ADDS**

- This study is the first to quantify differences in both primary and secondary care outcomes for COVID-19 and influenza in a UK regional population.
- Patients admitted for COVID-19 were more likely to die, and more likely to stay in hospital for over 10 days, than patients admitted for influenza.
- There were also higher levels of primary care activity recorded following discharge for patients with COVID-19 compared with those with influenza.
- Readmission rates were similar for both groups.

**HOW THIS STUDY MIGHT AFFECT RESEARCH, PRACTICE OR POLICY**

- Patients with COVID-19 interacted more with healthcare services and had poorer outcomes than those with influenza.
- The findings could inform postdischarge care planning for individuals recovering from acute COVID-19, and primary and secondary care resource planning, for example, winter planning.
- Quantification of the relative impact of COVID-19 may be relevant to future epidemics of viral respiratory infections.

## Introduction

Health systems internationally have struggled to cope with high numbers of acutely ill patients through the first and subsequent peaks of the COVID-19 pandemic. As well as the risk of severe disease and death, there are long-term health impacts for some individuals surviving the acute illness phase of COVID-19, and increasing evidence on the impact of this on patients and health services.^1 2^

Parallels have been drawn between COVID-19 and influenza due to assumed similarities in their clinical features. Initially, responses to the pandemic were based on planning for influenza epidemics. However, whereas influenza introduces a recurrent major challenge to healthcare systems and patient outcomes, the COVID-19 pandemic differs from recent seasonal influenza epidemics in its scale and global impact.^3^ The burden of the COVID-19 pandemic in terms of mortality, morbidity and the economic consequences, has been significantly greater than that associated with recent influenza epidemics.^4–6^ There are few studies examining differences in outcomes for patients and associated burden on health systems, accounting for differences in the demographic profiles of those with severe disease.^7 8^

In a review, patients with COVID-19 and influenza had many differences in clinical manifestations and radiographic findings.^9^ Another review^10^ reported that COVID-19 has a higher mortality compared with influenza with case fatality rate almost 15 times greater than that of influenza. However, these reviews summarised studies reporting on COVID-19 and influenza separately. Only a few studies reported on outcomes of COVID-19 versus influenza directly. Patients with COVID-19 had worse outcomes^7 11^ than patients with influenza, and had a higher mortality rate.^7 12 13^ Patients with COVID-19 had worse respiratory outcomes, including longer duration of mechanical ventilation compared with those with influenza,^7 12 13^ greater likelihood of stroke,^14^ acute respiratory distress syndrome, systemic inflammatory response syndrome or acute kidney injury^13^; longer hospitalisation and were admitted to ICU more often than patients with influenza.^15^

Patients with COVID-19 were more likely to be male,^12 13 16^ have a higher body mass index and have higher rates of chronic kidney disease and diabetes.^12^ They were typically younger, and healthier, with fewer comorbidities and lower medication use.^16^ In children, there was no difference in hospitalisation rates, yet more patients with COVID-19 reported clinical symptoms at the time of diagnosis.^17^

These studies provide evidence up to Spring 2020. Up to June 2020, the UK experienced some of the highest per capita mortality from COVID-19 of any country.^18^ It is therefore important to identify differences in the clinical presentation, patient demographics and prognosis of these two diseases. In particular, health system planning for future waves of COVID-19, and for subsequent winters, requires accurate predictions of the impact of both COVID-19 and influenza on service utilisation. Official statistics reported that the mortality rate for COVID-19 is also significantly higher than influenza for both 2020 and the 5-year average,^6^ and that people infected with both influenza and COVID-19 are more than twice as likely to die as someone with COVID-19 alone.^19^ Despite this, we are unaware of any peer-reviewed study that directly quantifies the difference between COVID-19 and influenza in patient outcomes and service utilisation for the two diseases in a UK population.

We therefore compared outcomes of patients admitted to hospital diagnosed with COVID-19 with patients admitted with a diagnosis of influenza in the previous 2 years, in the population of Northwest London.

## Methods

### Design and setting

We used a retrospective cohort study design to compare length of hospital stay, mortality, emergency readmission rate and interaction with primary care for patients admitted as an emergency with COVID-19 and influenza (table 1). The study took place in the geographical area of Northwest London. This area is covered by a National Health Service (NHS) Integrated Care System, comprising a single Clinical Commissioning Group, responsible for planning and commissioning healthcare services for the eight boroughs of Northwest London; nine provider Trusts providing hospital, mental health and community services and six local authority councils, responsible for provision of social care services and local public health services. The population of Northwest London is 2.4 million, with similar age-sex distribution to the UK as a whole, and higher ethnic diversity.^20^

**Table 1:** Outcome definitions

Outcome	Definition
Length of stay	Time difference in days between admission and discharge dates of the index admission. A binary variable was defined indicating patients whose index length of stay was in the upper quartile across both cohorts.
Readmission	A subsequent emergency admission for any cause, within 90 days of the index discharge date.
Mortality	For those who died outside hospital, we used the midpoint of their month of death as recorded in the Discover data. Binary outcome variables were defined for death in hospital, death prior to 30 and 90 days postdischarge (including in hospital).
Primary care activity	For each patient, the number of days in which at least one clinical code was assigned to their record. Details of the process by which administrative codes were removed from the data, to leave only clinical codes, are provided in online supplemental file 2.

10.1136/thoraxjnl-2021-217858.supp2 Supplementary data

### Data

We used the Northwest London Discover dataset, a large population-based dataset covering the region. This dataset comprises linked data on primary, secondary and tertiary care, community and mental healthcare, emergency departments and social care.^20^ The Discover dataset contains data on 2.3 million residents currently registered with a general practitioner, as well as 1.1 million previously registered, and 208 000 previous residents now deceased. Fully linked records are available from January 2015 to January 2021 inclusive. Primary care data are sourced from general practice electronic record systems, whereas secondary care data are sourced from a Secondary Uses Service (SUS) dataset.^21^

### Study population and definitions

The study consists of two cohorts: the COVID-19 cohort and the influenza cohort, both comprising adults registered with a general practitioner in Northwest London. Patients were assigned to the COVID-19 cohort if they were admitted to a Northwest London hospital at least once for COVID-19 during 1 February 2020 to 2 November 2020. Admission for COVID-19 was defined as a primary diagnosis International Classification of Diseases, Tenth Revision (ICD-10) code of U071. Patients were assigned to the influenza cohort if they were admitted to hospital at least once for influenza between 1 January 2018 and 31 December 2019, excluding those subsequently admitted for COVID-19. We chose the time period for the influenza cohort to be pre-COVID-19, since the epidemiology of influenza was affected by the COVID-19 pandemic. All emergency admissions to any acute hospital provider Trust in Northwest London were included. Influenza was defined as primary diagnosis ICD-10 codes J09, J10 or J11. For each patient, the index admission was defined as the first eligible admission. Patients with <90 days of follow-up available after index discharge were excluded; this comprised those whose index admission occurred <90 days prior to the end of the study.

Demographic variables were age group at index admission, sex, ethnic group and an Index of Multiple Deprivation determined using the patient’s lower super output area of residence.^22^

### Analysis

Patient characteristics and unadjusted outcomes were summarised. Differences between the cohorts were analysed using two-tailed Pearson’s χ^2^ tests, with significance level 0.05 and results expressed as p values and Cramer’s V statistic, a measure of effect size. Unadjusted ORs were calculated comparing the cohorts on each binary outcome, and the incidence rate ratio (IRR) similarly for number of primary care activity. Multivariable logistic regression models were fitted for each binary outcome variable, on cohort and adjusting for demographic covariates selected based on clinical expertise, and within the constraints of the available data (age group, sex, ethnic group, Index of Multiple Deprivation quintile). A negative binomial model was fitted for number of primary care activity. Patients with missing ethnicity or Index of Multiple Deprivation data were excluded from regression models, no other variables had missing data. We conducted three sensitivity analyses: to evaluate impact of excluding patients with insufficient follow-up time, to assess any bias due to demographic differences in the cohorts and a survival analysis for length of stay. Results of regression analyses are stated as ORs and IRRs, respectively, with 95% CIs. R statistical software V.3.6.0 was used for all analyses. While we did not undertake power calculations a priori, we have included them for context (see online supplemental file 1).

10.1136/thoraxjnl-2021-217858.supp1 Supplementary data

### Patient and public involvement

Lay members of the Northwest London Data Access Committee read the study synopsis and were part of a question-and-answer session before data access approval was granted. This work was also discussed with patient partners at a ‘problem solving’ session in May 2020. This work uses data provided by patients and collected by the NHS as part of their care and support.

### Role of the funding source

The National Institute for Health and Care Research had no role in the design and conduct of the study; collection, analysis or interpretation of data; the writing of the manuscript or the decision to submit it for publication.

## Results

**Figure 1:**
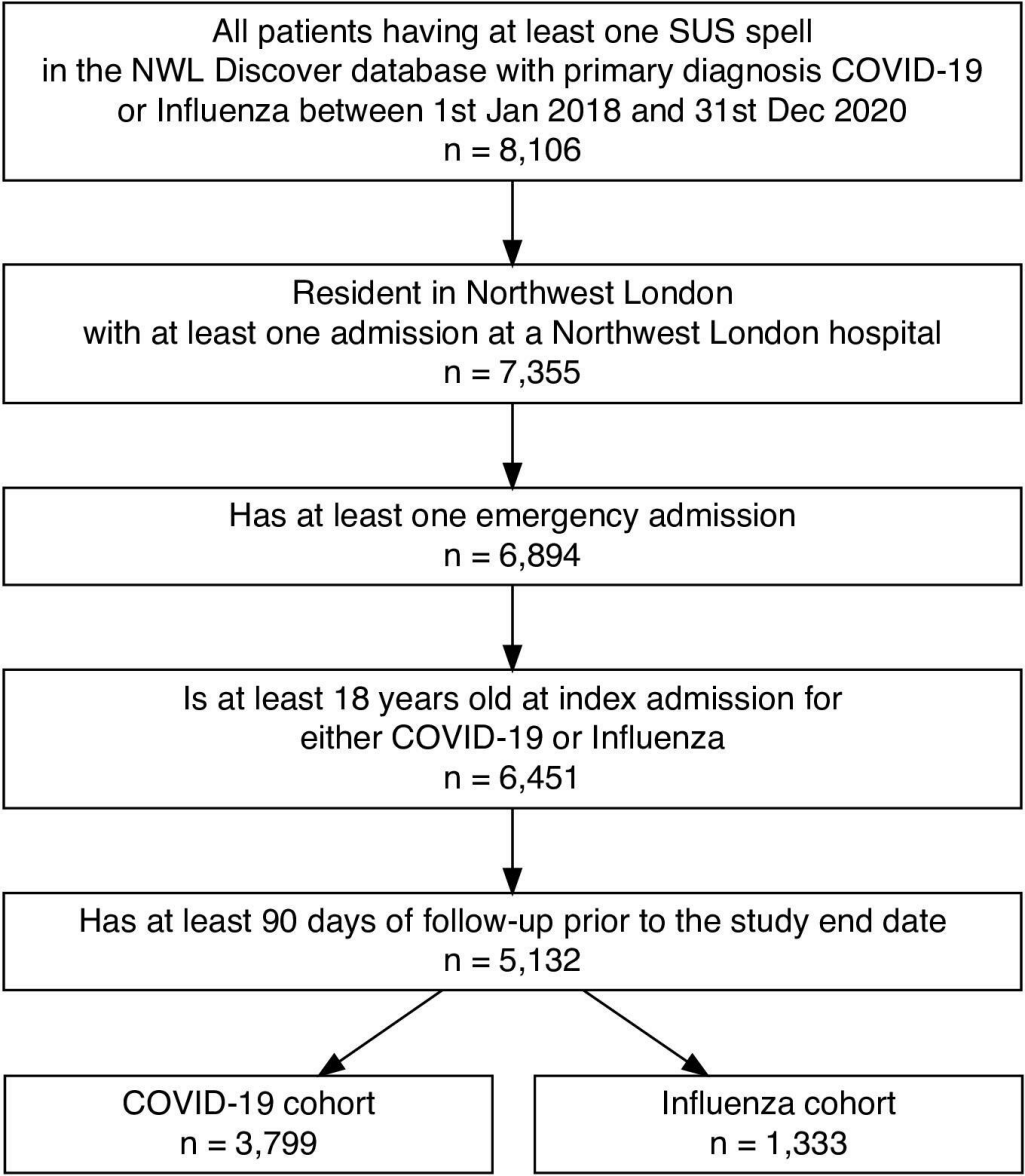
Study flow diagram. The Northwest London (NWL) Discover database contains all Secondary Uses Service (SUS) spells meeting either of the following criteria: (i) the patient is registered with a general practitioner in NWL or (ii) the SUS spell is for a hospital in NWL.

A total of 5132 patients met the inclusion criteria: 3799 (74.0%) in the COVID-19 cohort and 1333 (26.0%) in the influenza cohort (figure 1, table 2). This represents an annual incidence of 228 admissions per 100 000 population for COVID-19, 7.5 times that for influenza (30.3 per 100 000 population). The COVID-19 cohort were less likely than influenza admissions to be under 45 (9.7% vs 22.7%), corresponding to a small (V=0.19) but statistically significant difference in the overall age distribution (p<0.001). Patients with COVID-19 were more likely to be male than patients in the influenza cohort (61.4% vs 42.8%, p<0.001). While the difference in ethnicity between the cohorts was significant (p<0.001), with a higher proportion of the COVID-19 cohort of black ethnicity than in the influenza cohort (14.5% vs 9.5%), overall, the difference in ethnicities was small (V=0.07). There was no difference in the distribution of index of multiple deprivation between the cohorts (p=0.70, V=0.02).

**Table 2:** Demographic characteristics of patients with at least one emergency hospital admission for COVID-19 (1 February 2020–2 November 2020) or influenza (1 January 2018–31 December 2019)

	COVID-19: 3799	Influenza: 1333	Combined: 5132	χ^2^ p value, Cramer’s V
Age group (years) at index admission				P<0.001 V=0.19
18–34	141 (3.7%)	169 (12.7%)	310 (6.0%)	
35–44	228 (6.0%)	133 (10.0%)	361 (7.0%)	
45–54	537 (14.1%)	149 (11.2%)	686 (13.4%)	
55–64	691 (18.1%)	209 (15.7%)	900 (17.5%)	
65–79	1163 (30.6%)	351 (26.3%)	1514 (29.5%)	
80+	1039 (27.3%)	322 (24.2%)	1361 (26.5%)	
Sex				P<0.001 V=0.16
Male	2334 (61.4%)	570 (42.8%)	2904 (56.6%)	
Female	1465 (38.6%)	763 (57.2%)	2228 (43.4%)	
Ethnic group				P<0.001 V=0.07
White	1435 (37.8%)	544 (40.8%)	1979 (38.6%)	
Asian	1308 (34.4%)	481 (36.1%)	1789 (34.9%)	
Black	551 (14.5%)	127 (9.5%)	678 (13.2%)	
Mixed	114 (3.0%)	46 (3.5%)	160 (3.1%)	
Other	371 (9.8%)	128 (9.6%)	499 (9.7%)	
Missing	20 (0.5%)	7 (0.5%)	27 (0.5%)	
Index of Multiple Deprivation quintile				P=0.70 V=0.02
1 (most deprived)	712 (18.7%)	270 (20.3%)	982 (19.1%)	
2	1248 (32.9%)	434 (32.6%)	1682 (32.8%)	
3	1029 (27.1%)	335 (25.1%)	1364 (26.6%)	
4	490 (12.9%)	175 (13.1%)	665 (13.0%)	
5 (least deprived)	210 (5.5%)	77 (5.8%)	287 (5.6%)	
Missing	110 (2.9%)	42 (3.2%)	152 (3.0%)	

### Multivariable regression results

**Figure 2:**
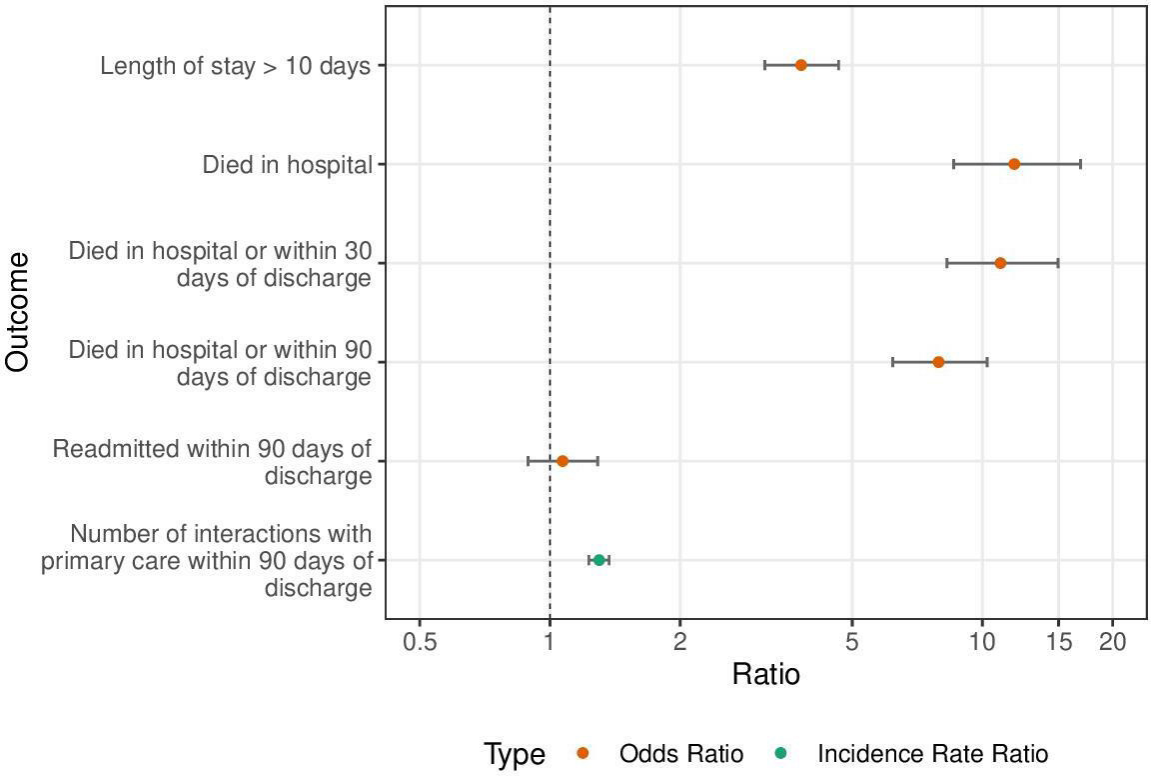
Outcomes for patients admitted to hospital as an emergency for COVID-19 (1 February 2020–2 November 2020) compared with influenza (1 January 2018–31 December 2019) in Northwest London. Each OR was derived from a multivariable logistic regression model for the respective outcome. The incidence rate ratio for primary care activity within 90 days of discharge was derived from a multivariable negative binomial regression model. All ratios are for COVID-19 cohort in comparison with the reference category influenza cohort, adjusted for age, sex, ethnic group and socioeconomic deprivation.

Patients with missing ethnicity or Index of Multiple Deprivation were excluded from multivariate analysis, as their numbers were too small to include an ‘unknown’ level for these variables in the models. For the mortality models, 179 of the total 5132 patients were excluded. For the other models, 122 of the 3802 patients who did not die during follow-up were excluded. Duration of index admissions was more often longer than 10 days (the overall upper quartile) for COVID-19 than influenza (adjusted OR (AOR) 3.81, 95% CI 3.14 to 4.65) (table 3, figure 2). Patients in the COVID-19 cohort were more likely to die during the index admission (AOR 11.85, 95% CI 8.58 to 16.86), between admission and 30 days postdischarge (AOR 11.01, 95% CI 8.28 to 14.96) and between admission and 90-day postdischarge (AOR 7.92, 95% CI 6.20 to 10.25). Among patients who did not die during index admission or 90 days postdischarge, rates of emergency readmission to hospital within 90 days were comparable between the two cohorts (AOR 1.07, 95% CI 0.89 to 1.29). However, primary care activity within 90 days of discharge was greater among the COVID-19 cohort (adjusted IRR 1.30, 95% CI 1.23 to 1.37). These relationships were qualitatively the same regardless of whether adjusted (table 3) or unadjusted (table 4) for patients’ demographic characteristics. Results of the sensitivity analyses (online supplemental file 1) did not qualitatively alter these results.

**Table 3:** Multivariable regression analysis results of various outcomes for patients admitted to hospital as an emergency for COVID-19 or influenza

Model outcome	Model 1: length of stay in upper quartile (>10 days)*† N=3680	Model 2: died in hospital N=4953	Model 3: died in hospital/in 30 days of discharge N=4953	Model 4: died in hospital/in 90 days of discharge N=4953	Model 5: readmitted within 90 days of discharge* N=3680	Model 6: primary care activity* N=3680
	Logistic regression models OR (95% CI), p value					Negative binomial model IRR (95% CI), p value
Cohort (influenza rc.)						
COVID-19	3.81 (3.14 to 4.65), p<0.001	11.85 (8.58 to 16.86), p<0.001	11.01 (8.28 to 14.96), p<0.001	7.92 (6.20 to 10.25), p<0.001	1.07 (0.89 to 1.29), p=0.48	1.30 (1.23 to 1.37), p<0.001
Age (18–54 rc.)						
55–64	1.63 (1.29 to 2.06), p<0.001	2.85 (2.07 to 3.97), p<0.001	3.07 (2.29 to 4.15), p<0.001	3.10 (2.34 to 4.15), p<0.001	1.48 (1.13 to 1.93), p=0.0045	1.29 (1.20 to 1.38), p<0.001
65–79	2.27 (1.84 to 2.81), p<0.001	6.90 (5.21 to 9.26), p<0.001	6.82 (5.27 to 8.94), p<0.001	6.67 (5.19 to 8.66), p<0.001	2.12 (1.68 to 2.69), p<0.001	1.47 (1.38 to 1.56), p<0.001
80+	4.40 (3.52 to 5.52), p<0.001	11.75 (8.86 to 15.82), p<0.001	12.26 (9.43 to 16.12), p<0.001	13.05 (10.11 to 17.02), p<0.001	2.75 (2.15 to 3.54), p<0.001	1.50 (1.41 to 1.61), p<0.001
Sex (male rc.)						
Female	0.94 (0.80 to 1.10), p=0.43	0.83 (0.71 to 0.97), p=0.022	0.79 (0.68 to 0.92), p=0.002	0.79 (0.68 to 0.91), p=0.0012	0.86 (0.72 to 1.02), p=0.090	1.07 (1.02 to 1.12), p=0.010
Ethnicity (white rc.)						
Asian	0.70 (0.59 to 0.85), p=0.00020	1.07 (0.89 to 1.28), p=0.47	1.08 (0.91 to 1.28), p=0.40	1.08 (0.91 to 1.28), p=0.38	0.96 (0.79 to 1.18), p=0.71	1.02 (0.96 to 1.08), p=0.49
Black	0.88 (0.68 to 1.12), p=0.30	1.11 (0.87 to 1.41), p=0.40	1.11 (0.88 to 1.40), p=0.38	1.08 (0.86 to 1.36), p=0.50	0.95 (0.72 to 1.25), p=0.72	1.01 (0.93 to 1.09), p=0.82
Mixed	0.76 (0.47 to 1.19), p=0.24	0.95 (0.57 to 1.54), p=0.85	0.98 (0.61 to 1.55), p=0.94	1.04 (0.65 to 1.61), p=0.87	0.57 (0.30 to 0.99), p=0.062	0.93 (0.81 to 1.07), p=0.28
Other	0.81 (0.61 to 1.07), p=0.14	1.10 (0.82 to 1.45), p=0.52	0.984 (0.748 to 1.29), p=0.91	0.98 (0.75 to 1.27), p=0.87	0.76 (0.55 to 1.05), p=0.10	0.92 (0.85 to 1.00), p=0.06
IMD quintile (1—most deprived rc.)						
2	0.92 (0.74 to 1.15), p=0.47	1.04 (0.84 to 1.29), p=0.72	1.07 (0.87 to 1.32), p=0.54	1.06 (0.87 to 1.31), p=0.56	0.97 (0.76 to 1.23), p=0.77	0.96 (0.90 to 1.03), p=0.30
3	0.96 (0.76 to 1.20), p=0.71	0.76 (0.61 to 0.96), p=0.022	0.87 (0.70 to 1.08), p=0.21	0.88 (0.71 to 1.09), p=0.25	0.84 (0.65 to 1.08), p=0.17	0.96 (0.90 to 1.03), p=0.28
4	0.83 (0.63 to 1.10), p=0.19	0.85 (0.64 to 1.11), p=0.23	0.90 (0.69 to 1.17), p=0.43	0.84 (0.65 to 1.09) p=0.19	0.79 (0.58 to 1.08), p=0.14	0.97 (0.89 to 1.06), p=0.48
5—least deprived	1.07 (0.74 to 1.54), p=0.72	0.92 (0.64 to 1.30), p=0.63	1.01 (0.72 to 1.42), p=0.94	1.04 (0.75 to 1.45), p=0.81	0.58 (0.36 to 0.90), p=0.018	0.96 (0.86 to 1.08), p=0.51

Admissions were to hospitals in Northwest London, between 1 February 2020 and 2 November 2020 for the COVID-19 cohort and between 1 January 2018 and 31 December 2019 for the influenza cohort. Regression model output is shown as ORs for binary outcomes, and IRRs for primary care activity, with 95% CI and p value. The reference category for each covariate is denoted by rc. Ratios for all terms in the regression models are reported in this table. No interaction terms were included.

*Readmissions, length of stay and primary care activity are evaluated only for patients who did not die in hospital or within 90 days of discharge.

†Comparison for length of stay is Q4 vs Q1–3.

IMD, Index of Multiple Deprivation; IRR, incidence rate ratio.

**Table 4:** Univariate and descriptive analysis of various outcomes for patients admitted to hospital as an emergency for COVID-19 or influenza

	COVID-19	Influenza	Combined	Unadjusted OR/IRR (95% CI), p value: COVID-19 compared with influenza
Length of stay in days*	N=2546	N=1256	N=3802	
Median: (LQ–UQ)	7 (3–12)	3 (1–6)	5 (2–10)	
Quartile: n (%)				
Q1: <2	479 (18.8%)	513 (40.8%)	992 (26.1%)	
Q2: 3–5	605 (23.8%)	369 (29.4%)	974 (25.6%)	
Q3: 6–10	699 (27.5%)	238 (18.9%)	937 (24.6%)	
Q4: >10	763 (30.0%)	136 (10.8%)	899 (23.6%)	3.52 (2.89 to 4.32)†
Mortality:	N=3799	N=1333	N=5132	
Died in hospital	1025 (27.0%)	39 (2.9%)	1064 (20.7%)	12.26 (8.84 to 17.46), p<0.001
Died in hospital or within 30 days of discharge	1196 (31.5%)	52 (3.9%)	1248 (24.3%)	11.32 (8.50 to 15.36), p<0.001
Died in hospital or within 90 days of discharge	1253 (33.0%)	77 (5.8%)	1330 (25.9%)	8.03 (6.31 to 10.34), p<0.001
Readmission*: n (%)	N=2546	N=1256	N=3802	
Readmitted within 90 days of discharge	459 (18.0%)	209 (16.6%)	668 (17.6%)	1.10 (0.92 to 1.33), p=0.29
Primary care activity*	N=2546	N=1256	N=3802	
Primary care activity: number of distinct days with clinical coding	27 256 (mean 10.7 per patient)	10 466 (mean 8.3 per patient)	37 722 (mean 9.9 per patient)	IRR 1.28 (1.26 to 1.31), p<0.001

Admissions were to hospitals in Northwest London, between 1 February 2020 and 2 November 2020 for the COVID-19 cohort and between 1 January 2018 and 31 December 2019 for the influenza cohort. Univariate comparison results are ORs for binary outcomes, and IRRs for primary care activity, with 95% CIs and p values.

*Readmissions, length of stay and primary care activity are evaluated only for patients who did not die in hospital or within 90 days of discharge.

†Comparison for length of stay is Q4 vs Q1–3.

IRR, incidence rate ratio.

## Discussion

### Summary of main findings

Using a linked, population-level dataset, we found a 7.5 times higher incidence of acute hospital admission for COVID-19 during the first wave of infection in 2020 than for influenza during 2018–19. Those admitted for COVID-19 were more likely to be older and male than those admitted for influenza. A greater proportion of patients were of black ethnicity in the COVID-19 cohort. After adjusting for demographics, patients admitted with COVID-19 were much more likely to die in hospital or to stay in hospital for over 10 days, and to die within 30 or 90 days of discharge, compared with those admitted for influenza. Patients who did not die were equally likely to be readmitted to hospital within 90 days of discharge for the two diseases, but the COVID-19 cohort interacted with primary care services more than the influenza cohort.

### Correspondence with current literature

This study offers new direct evidence of longer hospital stays for COVID-19 admissions compared with influenza. Our findings of greater activity by primary care services among the COVID-19 cohort, but no difference in emergency readmission rates, are new. The higher mortality rates seen among patients admitted with COVID-19 compared with influenza are in accordance with existing evidence from France and the USA.^7 8^ Moreover, our study adds to the growing body of knowledge internationally concerning the impact of severe COVID-19 infection for patients and for health service providers, including primary care as well as specialist providers.

Our study confirms that COVID-19 has a bigger impact on health outcomes and service use than influenza at both an individual and a population level. Factors contributing to this include the likely higher potential of COVID-19 for respiratory pathogenicity, leading to more respiratory complications, greater risk of ischaemic stroke and higher mortality.^7 14^ This reinforces the need for effective prevention measures to limit the spread and impact of COVID-19, including non-pharmaceutical interventions, as well as vaccination.^23^

### Policy implications

Our findings have implications for enhanced pandemic response planning, in terms of understanding patient and service outcomes in relation to influenza, a disease that is currently better understood than COVID-19 and which health services have years of experience dealing with. First, the results on service use both for secondary and primary care during the COVID-19 pandemic have implications for care planning for individuals being discharged from hospital having recovered from acute COVID-19. For example, many patients will need ongoing support in primary care after discharge. Second, our findings provide data for improved service planning, for primary care and perhaps most significantly for secondary care resource planning, for example, in NHS winter planning. Existing scenarios based on influenza epidemic planning may be adapted, taking into account the relative scale of service use for COVID-19 identified in this study. As well as providing an evidence base for such planning in the near future, this quantification of the relative impact of COVID-19 may be of use in responding to future epidemics of viral respiratory infections.

Similar 90-day emergency readmission rates for the two diseases may indicate that learning from interventions used to avoid hospital admissions during the COVID-19 pandemic could potentially be applied to future seasonal influenza epidemics.

### Strengths and limitations

This study directly compared patient and service use outcomes following admission to hospital for COVID-19 and influenza. Unlike comparisons made with the general population, this offers insights into differences between disease trajectories independently of other factors associated with hospital admission. We used hospital diagnoses, rather than test results, to define the cohorts, thus avoiding bias due to less comprehensive testing for influenza than COVID-19 during the study period. The diverse population-wide scope of the Discover data virtually eliminated the risk of selection bias due to data coverage, and the routinely collected nature of the data meant that data were available for every patient admitted and coded for the two diseases over the study period. The only exceptions to this were hospital admissions occurring outside of Northwest London, but this was the same for both cohorts so is not likely to have affected the findings. The datasets used have been found to have good face validity compared with established research datasets.^20^ Although generalisations from these study findings to other populations should be made with caution, previous research has shown that the study population matches the overall age-sex and chronic disease prevalence distribution of the UK well, while being more ethnically diverse. The SUS data, and the derived hospital episode statistics, are a well-established source of data for observational studies in England.^24^ A systematic review found that the accuracy of clinical coding used in such databases is sufficiently robust for research purposes.^25^ Taken together, these features convey significant advantages in terms of the validity of this study, strengthening the potential for our results to inform policy.

The main limitations of this study are common among similar studies based on routinely collected data. The quality of data recorded was dependent on healthcare provider practices in relation to diagnosis and clinical coding. Date of death was only available to the nearest month for deaths outside of hospital, this was the same for both cohorts. Data on consultations with general practitioners were not available, so we instead considered clinically read-coded interactions with primary care as a proxy for primary care activity. While this likely overestimated actual use of services by patients, this approach was also the same across both cohorts. We cannot rule out systematic changes in coding between the two cohorts however, and future research should seek to explore the nature and extent of primary care use among people discharged from hospital following an acute episode of COVID-19. The covariates included in our models were chosen to account for the difference in demographic profiles of both incidence and disease severity for COVID-19 and influenza, however, we did not have data on infection severity and so could not adjust for this independently. The Index of Multiple Deprivation is established at a local geography level, each area covering between 1000 and 3000 individuals. While this is not as precise as an individual level measure of socioeconomic deprivation, this was the most precise measure available in the data and is commonly used in observational studies. While other potential sources of confounding exist, such as prior disease history, there were not enough admissions to include these variables in our regression models. Similarly, there were insufficient admissions in these data to explore interaction effects between demographics and the disease cohort. Future research should explore this relationship, especially from the perspective of health inequalities.

The study period did not include winter admissions for COVID-19, so it was not possible to adjust for seasonality in the models. Influenza is highly variable in pathogenicity year on year, and the years chosen (2018 and 2019) may be atypically mild or severe. The COVID-19 pandemic placed huge stress on health service provision during the study period (in 2020), which was not present to the same degree in the years used for comparison with influenza. COVID-19 outcomes have, overall, improved as the pandemic has progressed, therefore the reported comparisons may differ for subsequent waves and variants of COVID-19. In this study, we focused on straightforward binary and count outcomes within a fixed follow-up period posthospital discharge. We excluded patients who died during follow-up from analyses of the other outcomes, providing a perspective on postdischarge service use given survival, rather than an assessment of these outcomes with death as a competing risk.

## Conclusions

Patients admitted to hospital as an emergency for COVID-19 are more likely to die in hospital and within 90 days of discharge from hospital, more likely to stay in hospital for >10 days and interact more with primary care after discharge than patients admitted for influenza. However, readmission rates within 90 days are similar for the two diseases among those who survive. These findings provide insight into COVID-19 outcomes in comparison to influenza, a more familiar disease. Health service commissioners and providers should draw on the findings of this study, specifically the impact of increased length of stay and primary care activity, in planning and providing services during the pandemic, during winter and in future epidemics. This is likely to be particularly important should COVID-19 become endemic and seasonal, like influenza.

## Data Availability

Data may be obtained from a third party and are not publicly available. The Discover data that support the findings of this study are available from Imperial College Partners, but restrictions apply to the availability of these data, which were used under licence for the current study, and so are not publicly available. Researchers wishing to access Discover data can apply as described by Bottle *et al.* ^20^
