# Supplementary material for: Patient outcomes following emergency admission to hospital for COVID-19 compared with influenza: retrospective cohort study
Source: Thorax. 2022 Jul 27;78(7):706–12. doi: 10.1136/thoraxjnl-2021-217858 (PMC10314037; doi:10.1136/thoraxjnl-2021-217858)
Supplement: Supplementary data [file thoraxjnl-2021-217858supp002.pdf]

Data supplement 2

Read codes excluded from patient records when counting distinct days on which primary care clinical activity was recorded.

As a proxy for primary care activity in relation to patients in this study, we counted the number of days in a 90 day period following discharge from hospital. To avoid counting days on which only administrative activity occurred, we used the clinical codes table provided by the Discover database, which first removes any Read CTV2 codes from the data that pertain to purely administrative functions.

The full detail of this process is provided by the SQL code below.

```
Select ReadCodeV2 collate
SQL_Latin1_General_CP1_CS_AS,ReadCodePreferredTerm30
          From
          DimReadCodeV2
          where
          (ReadCodeV2 like '9%' -- Remove for Admin activity
from costing DZ 08/2017
               and not ReadCodeV2 collate
SQL_Latin1_General_CP1_CS_AS  in (
     '9b0m.','9b0n.','9bE5.','9k27.','9l0..','9m40.','9m42.','
9m43.','9N2q.',

     '9N2R.','9N2r.','9N2s.','9N2S.','9N2T.','9N2t.','9N2u.','
9N2U.','9N2V.','9N2v.',

     '9N2w.','9N2W.','9N2X.','9N2x.','9N2Y.','9N2y.','9N2z.','
9N3..','9N31.','9N311',

     '9N34.','9N3A.','9N3F.','9N45.','9N46.','9N4P.','9N5..','
9N58.','9N5B.','9N79.',

     '9N7A.','9N7E.','9N7F.','9N7G.','9Na1.','9NzF.','9OX50','
9OX51','9OX52','9OX54',
     '9OX55','9OX56','9OX57','9Ed..','9H9..','9H90.','9H91.','
9H92.')) -- Phase 2 bringing back some read codes
          or ReadCodeV2 collate SQL_Latin1_General_CP1_CS_AS
in ('9N2..', '9N2Z.')
          or ReadCodeV2 collate SQL_Latin1_General_CP1_CS_AS
in (
     '4141.','4142.','4143.','4144.','4145.','4146.','4147.','
4149.',
     '4214.','4411.','4615.','0....','134..','414..','419..','
41B1.',
     '41B10','41B2.','41C1.','44H1.','44I1.','44O1.','46D1.','
4J22.',
     '4J41.','4JF5.','4JJ1.','4JJ2.','4JJ3.','4K11.','4K12.','
67DJ.',
```

```
      '681C.','685M.','8B3h.','8B3U.','8B4..','8B41.','8BM8.','
8BMA.',

      '8BMC.','8BME.','8BMG.','8C1B.','8CE5.','8H2..','8HE..','
8MD..',
                 'ZV68.','ZV681','38Gt0')
```
