# Supplementary material for: Patient outcomes following emergency admission to hospital for COVID-19 compared with influenza: retrospective cohort study
Source: Thorax. 2022 Jul 27;78(7):706–12. doi: 10.1136/thoraxjnl-2021-217858 (PMC10314037; doi:10.1136/thoraxjnl-2021-217858)
Supplement: Supplementary data [file thoraxjnl-2021-217858supp001.pdf]

Data supplement 1

1 Statistical Power

We did not undertake a power calculation in advance for this study. However, for context we include here power calculations performed after the study was complete.

We performed power calculations for logistic regression based on one covariate, cohort.(1) All power calculations use a type I error rate of 5%. For the logistic regression models, we have not used the effect sizes observed in this study, since this simply yields a one-to-one function of the reported p-value, but rather have drawn on a priori literature where available to derive minimum detectable effect sizes.(2) Baseline odds were derived from the observed outcomes for influenza however, as was the cohort balance.

We used the odds ratio for in hospital mortality from Piroth et al. as the minimum detectable effect size for all mortality outcomes.(3) For readmissions we used the readmission rate for COVID-19 from Donnelly et al.(4) For long length of stay we used odds ratios of 2 and 1.5, as no literature was available a priori for this outcome. For the primary care events outcome, we calculated power for negative binomial regression using the observed data from this study, since no comparable literature was available a priori. For this we used the maximum likelihood approach.(5) The resulting minimum sample sizes to achieve power of 80% are shown in the table below.

Outcome	Sources	Baseline (influenza) proportion/rate	Proportion/rate (COVID-19 minimum detectable)	Minimum detectable Odds/Rate Ratio	Cohort Balance (% COVID-19)	Required sample size
In-hospital mortality	Piroth et al.	2.9%	20.7%	8.72	74%	297
30 day mortality	Piroth et al.	3.9%	26.1%	8.72	74%	226
90 day mortality	Piroth et al.	5.8%	34.9%	8.72	74%	158
90 day readmission	Donnelly et al.	16%	25.0%	1.75	67%	912
Long length of stay	-	10.8%	19.5%	2	67%	802
Long length of stay	-	10.8%	15.4%	1.5	67%	2,348
Primary care events	This study	8.3	10.6	1.28	67%	374

We also computed the power that would be achieved for these effect sizes, with the actual number of observations in this study, shown in the table below.

Outcome	Sources	Baseline (influenza) proportion/rate	Proportion/rate (COVID-19 minimum detectable)	Minimum detectable Odds/Rate Ratio	Cohort Balance (% COVID-19)	Actual sample size	Power
In-hospital mortality	Huang et al.	2.9%	20.7%	8.72	74%	5,132	99.9%
30 day mortality	Huang et al.	3.9%	26.1%	8.72	74%	5,132	99.9%
90 day mortality	Huang et al.	5.8%	34.9%	8.72	74%	5,132	99.9%
90 day readmission	Donnelly et al.	16%	25.0%	1.75	67%	3,802	99.9%
Long length of stay	-	10.8%	19.5%	2	67%	3,802	99.9%
Long length of stay	-	10.8%	15.4%	1.5	67%	3,802	94.9%
Primary care events	This study	8.3	10.6	1.28	67%	3,802	99.9%

These calculations give some reassurance that the study was powered to detect differences between the influenza and COVID-19 on the level described in the literature, and indeed on the level identified in this study.

2 Sensitivity Analyses

2.1 Sensitivity analysis 1: follow-up time

Aim of sensitivity analysis
To understand the potential impact of any bias introduced through excluding patients with less than 90 days of follow-up

Methods
The 1,319 patients excluded through insufficient follow-up time were those whose index admission date was less than 90 days prior to the study end date (2nd November 2020). To emulate the impact of this exclusion, we re-ran the main regression analyses on a subset of the study population formed by setting the study end date to an earlier date, such that as close as possible to 1,319 were further excluded. This end date for the sensitivity analysis was 11th July 2020, resulting in exclusion of a further 1,262 patients from the COVID-19 cohort.

Results

The influenza cohort was unchanged from the main analysis, comprising the same 1,333 patients. The COVID-19 comprised 2,537 patients, compared with 3,799 in the main analysis. Of the total 3,870 patients included in the sensitivity analysis, 136 had missing ethnicity or IMD data and were excluded from the regression models, as in the main analysis. Of the 2,841 patients who did not die during the follow up period, and were hence included in the length of stay, readmission and primary care interaction models, there were 88 excluded due to missing data. Odds ratios of each outcome for COVID-19 compared with Influenza are shown in supplementary table 1.

**Outcome**		**Full Study (as per table 4 in main manuscript)**	**Sensitivity Analysis**
**Model 1: Length of stay in upper quartile (> 10 days)**	Logistic regression models OR (95% CI), p	3.81 (3.14 to 4.65)<br>p < 0.001<br>N = 3,680	4.31 (3.496 - 5.34)<br>p < 0.001<br>N = 2,753
**Model 2: Died in hospital**		11.85 (8.58 to 16.86)<br>p < 0.001<br>N = 4,953	15.4 (11.1 - 22.0)<br>p < 0.001<br>N = 3,734
**Model 3: Died in hospital / in 30d of discharge**		11.01 (8.28 to 15.0)<br>p < 0.001<br>N = 4,953	14.2 (10.6 - 19.4)<br>p < 0.001<br>N = 3,734
**Model 4: Died in hospital / in 90 days of discharge**		7.92 (6.20 to 10.25)<br>p < 0.001<br>N = 4,953	10.0 (7.81 - 13.1)<br>p < 0.001<br>N = 3,734
**Model 5: Readmitted within 90 days of discharge**		1.07 (0.89 to 1.29)<br>p = 0.48<br>N = 3,680	0.976 (0.792 - 1.21)<br>p = 0.82<br>N = 2,753
**Model 6: Number of interactions with primary care**	Negative binomial IRR (95% CI), p	1.30 (1.23 to 1.37)<br>p < 0.001<br>N = 3,680	1.26 (1.19 - 1.33)<br>p < 0.001<br>N = 2,753

2.2 Sensitivity analysis 2: differences between cohorts

Aim of sensitivity analysis

To assess the extent to which the main findings might be biased by differences in demographics between the two cohorts

Methods

We conducted sensitivity analyses by creating matched cohorts of influenza and COVID-19 patients, using exact matching on all covariates in the main analyses; namely age band, sex, ethnic group, and index of multiple deprivation quintile. For each outcome measure we then fitted a weighted univariate regression model, of the same type as for the main analysis, with weights derived from the matching procedure. Model results are provided as marginal odds ratios or incidence rate ratios for the COVID-19 cohort compared with the influenza cohort. 95% confidence intervals were calculated using cluster-robust standard errors.

Results

After matching there were 4,864 patients eligible for inclusion in the mortality analyses, and 3,620 in the other analyses. The results were very similar to those of the main analyses, with all effects in the same direction, of the same statistical significance, and of comparable magnitudes. These results provide reassurance that the main findings are not adversely affected by differences in the observed demographics of the two cohorts.

**Outcome**	**Model 1: Length of stay in upper quartile (> 10 days)**	**Model 2: Died in hospital**	**Model 3: Died in hospital / in 30d of discharge**	**Model 4: Died in hospital / in 90 days of discharge**	**Model 5: Readmitted within 90 days of discharge**	**Model 6: Number of interactions with primary care**
N (before matching)	**3,680**	**4,953**	**4,953**	**4,953**	**3,680**	**3,680**
n (after matching)	3,620	4,864	4,864	4,864	3,620	3,620
	Logistic regression models OR (95% CI), p-value					Negative binomial regression model IRR (95% CI), p-value
Cohort (Influenza rc.) COVID-19	3.38 (2.81 to 4.09), p < 0.001	9.19 (6.22 to 13.56), p < 0.001	8.88 (6.28 to 12.55), p < 0.001	6.67 (5.33 to 8.44), p < 0.001	1.11 (0.90 to 1.36), p = 0.26	1.26 (1.19 to 1.32), p < 0.001

2.3 Sensitivity Analysis 3: Survival analysis for length of stay in hospital

Aim of sensitivity analysis

Survival analysis offers an alternative, and potentially more sensitive, approach to comparing length of stay for COVID-19 and influenza, and as such we conducted a sensitivity analyses, censoring deaths before successful hospital discharge, as an additional check of the robustness of the main findings for this outcome.

Methods

Using the same inclusion and exclusion criteria as for the main regression models, we first fitted a Cox proportional hazard model, and assessed compliance with the proportional hazards assumption graphically and using Schoenfeld residuals. Where the proportional hazards assumption did not hold we fitted accelerated failure time models, for a range of different distributions, and assessed the accelerated failure assumption graphically. We also fitted Cox models with time-varying coefficients where indicated.

Results

There were n = 4,953 patients included in the analysis. The proportional hazards assumption did not hold (overall Schoenfeld test $p < 0.001$), being violated for the main variable of interest, namely cohort, and for age band. Stratification by age band still left proportional hazards violated for cohort ($p < 0.001$). Model assumptions were also violated for all accelerated failure time models fitted (exponential, Weibull, lognormal and log-logistic distributions), and for Cox models with time-varying coefficients for cohort and age band.

The table shows the hazard ratios and p-values for the Cox proportional hazard model stratified by age band; it should be noted that since the proportional hazards assumption is violated here, the hazard ratio for cohort may only be interpreted as an average effect over the timepoints observed in the data.(6) The average hazard ratio for cohort (0.44, 95% CI 0.41 to 0.47) is consistent with the main analysis results of longer lengths of stay in the COVID-19 cohort on average.

Variable	Hazard Ratio (95% CI)	p-value
COVID-19	0.46 (0.43 to 0.50)	p < 0.001
Sex (Male rc.)		
Female	1.09 (1.02 to 1.16)	p = 0.011
Ethnic Group (White rc.)		
Asian	1.14 (1.06 to 1.23)	p < 0.001
Black	1.06 (0.95 to 1.17)	p = 0.307
Mixed	1.21 (1.01 to 1.45)	p = 0.037
Other	1.13 (1.01 to 1.27)	p = 0.032
IMD Quintile (1 – most deprived rc.)		
2	1.03 (0.94 to 1.13)	p = 0.503
3	1.08 (0.98 to 1.18)	p = 0.122
4	1.15 (1.03 to 1.29)	p = 0.014
5 – least deprived	1.05 (0.90 to 1.22)	p = 0.565

References

1. Hsieh FY, Bloch DA, Larsen MD. A simple method of sample size calculation for linear and logistic regression. Statist Med. 1998 Jul 30;17(14):1623–34.

2. Hoenig JM, Heisey DM. The Abuse of Power: The Pervasive Fallacy of Power Calculations for Data Analysis. The American Statistician. 2001 Feb;55(1):19–24.

3. Piroth L, Cottenet J, Mariet AS, Bonniaud P, Blot M, Tubert-Bitter P, et al. Comparison of the characteristics, morbidity, and mortality of COVID-19 and seasonal influenza: a nationwide, population-based retrospective cohort study. The Lancet Respiratory Medicine. 2021 Mar;9(3):251–9.

4. Donnelly JP, Wang XQ, Iwashyna TJ, Prescott HC. Readmission and Death After Initial Hospital Discharge Among Patients With COVID-19 in a Large Multihospital System. JAMA. 2021 Jan 19;325(3):304.

5. Zhu H, Lakkis H. Sample size calculation for comparing two negative binomial rates. Statist Med. 2014 Feb 10;33(3):376–87.

6. Allison PD. Survival analysis using SAS: a practical guide. 2. ed. Cary, NC: SAS Press; 2010. 324 p.
